# Clinical Relevance of Body Fluid Volume Status in Diabetic Patients With Macular Edema

**DOI:** 10.3389/fmed.2022.857532

**Published:** 2022-04-12

**Authors:** Jie Yao, Qingsheng Peng, Yuanhong Li, Anyi Liang, Jianteng Xie, Xuenan Zhuang, Ruoyu Chen, Yesheng Chen, Zicheng Wang, Liang Zhang, Dan Cao

**Affiliations:** ^1^Department of Ophthalmology, Guangdong Provincial People's Hospital, Guangdong Academy of Medical Sciences, Guangzhou, China; ^2^Shantou University Medical College, Shantou, China; ^3^Department of Nutrition, Guangdong Provincial People's Hospital, Guangdong Academy of Medical Sciences, Guangzhou, China; ^4^Department of Nephrology, Guangdong Provincial People's Hospital, Guangdong Academy of Medical Sciences, Guangzhou, China; ^5^State Key Laboratory of Ophthalmology, Zhongshan Ophthalmic Center, Sun Yat-sen University, Guangzhou, China; ^6^Southern Medical University, Guangzhou, China; ^7^Department of Cardiology, Guangdong Cardiovascular Institute, Guangdong Provincial People's Hospital, Guangdong Academy of Medical Sciences, Guangzhou, China; ^8^School of Medicine, South China University of Technology, Guangzhou, China

**Keywords:** diabetic macular edema (DME), optical coherence tomography, body composition measurement, fluid status, albuminuria

## Abstract

**Objective:**

To investigate body fluid status in diabetic macular edema (DME) patients and the extent to which it is affected by renal function.

**Methods:**

One hundred and thirty-two eyes from 132 patients with diabetes mellitus (DM) were prospectively collected in this cross-sectional, observational study. Thirty-five were DM patients without diabetic retinopathy (DR), 31 were DR patients without DME, and 66 were DME patients. The fluid status of each participant was quantified with extracellular water-to-total body water ratio (ECW/TBW) using a body composition monitor. Central subfield thickness (CST) and macular volume (MV) were obtained using optical coherence tomography (OCT). Urine albumin-to-creatinine ratio (UACR), estimated glomerular filtration rate (eGFR), and albumin was obtained using serum and urine laboratory data.

**Results:**

ECW/TBW was significantly increased in DME patients (39.2 ± 0.9, %) compared to DM (38.1 ± 0.7, %, *P* = 0.003) and DR patients without DME (38.7 ± 0.9, %, *P* < 0.001). In multilinear regression, fluid overload was positively related to DME and UACR (DME vs. DM: β = 2.418, *P* < 0.001; DME vs. DR: β = 1.641, *P* = 0.001; UACR, per 10^2^, β = 1.017, *P* = 0.01). In the binary logistic regression for DME risk, the area under the receiver operating characteristic curve (AUROC) increased significantly by adding ECW/TBW along with UACR and age (AUC: 0.826 vs. 0.768).

**Conclusion:**

DME patients had elevated body fluid volume independent of kidney functions. The assessment of extracellular fluid status may help in the management of DME.

## Introduction

The burden of diabetes mellitus (DM) is growing globally, with a worldwide estimated prevalence of 4.1% in 2010 expected to increase to 7.7% by 2030 (1, 2). In China, up to 11.6% of the population aged 18 years and older has diabetes (3). Diabetic microvascular complications such as retinopathy (DR) and macular edema (DME) are the major causes of blindness in DM patients in China (4). Diabetic kidney disease (DKD) is another common microvascular complication that leads to kidney failure in approximately 40% of diabetic patients (5). Our group found that the elevation of urine albumin-to-creatinine ratio (UACR) or the worsening in UACR stages, a signature change for renal function in DKD, was correlated with DME (6) and macular thickening (7). Clinical evidence also supports this connection. DME patients with better renal function had greater chances of better prognoses, especially in refractory DME (8, 9). DKD and DR share similar pathogenesis (10). Hyperglycemia-induced inflammation and oxidative stress lead to loss of endothelial cells, pericytes, and disruption of cell junctions, which contribute to the breakdown of vascular barrier in both the kidney and retina (10). For DME, hyperglycemia-linked pathways also contribute to dysfunction of retinal pigment epithelium (RPE) cells and glial cells such as Müller cells, which normally drain the fluid in the retina to the systemic circulation and keep the retina dehydrated (11, 12). Increased entry of fluid through the damaged blood-retinal barrier (BRB) and decreased drainage result in accumulation of fluid, especially in the macula (13).

Interestingly, while DKD has been proven associated with the elevation in extracellular fluid volume (14), a recent study discovered that fluid overload also appeared in DME patients (15). Imbalance of hydrostatic and oncotic pressure explained by starling equation could account for the phenomenon. The fluid status was monitored by body composition measurement (BCM) objectively and non-invasively, which is widely assumed credible assessing the fluid status of patients with chronic kidney diseases in clinical practice (16, 17). DME characterized by exudative fluid accumulation in the macula was speculated as a focal manifestation of extracellular fluid overload. The accessibility of extracellular water to total body water (ECW/TBW) ratio makes it the perfect systemic fluid marker for DME studies and a potential biomarker for DME treatments (18). Still, the confounding effect between DKD and fluid overload has not been discussed deeply regarding the relationship with DME. There lacked evidence of whether the fluid expansion came up through DME pathophysiological alterations along or shared pathogenesis between DME and DKD.

In this paper, the fluid statuses among different DM patients, including DR patients with or without DME and diabetic patients with or without DR, were investigated. By further exploiting associated changes of fluid status and renal function parameters in DME, this study proposed a novel hypothesis for the role of body fluid status in DME mechanisms. It provided significant clinical evidence to guide future DME management and treatments.

## Methods

This was a cross-sectional observational study. Diabetic patients diagnosed with or without DR and DME by a multi-disciplinary team at Guangdong Provincial People's Hospital were prospectively included from May 2020 to February 2021. Medical records were reviewed by a chief-resident doctor from the department of ophthalmology and a chief-resident doctor from the department of endocrine of Guangdong Provincial People's Hospital. All patients included were informed of the study contents and signed written consent. This study was conducted following the Declaration of Helsinki and under the supervision of the Research Ethics Committee of Guangdong Provincial People's Hospital, Guangdong Academy of Medical Sciences (registration number: GDREC2018380H).

### Participant

Patients aged 18 years or older, diagnosed with DM, with or without DR and DME, and capable of going through funduscopic examination, optical coherence tomography (OCT) scans, and BCM were included in the study. Exclusion criteria were receiving pan-retinal photocoagulation, intravitreal injection or pars-planar vitrectomy within 3 months, other retinal diseases including age-related macular disease, retinal vascular occlusion, retinal vasculitis, macular hole, and epiretinal membrane, significant cataract affecting fundus examination, and severe systemic diseases including myocardial infarction, stroke, CKD at end-stage, and under hemodialysis or peritoneal dialysis.

### Clinical Parameters

All patients were examined for the following characteristics and parameters: age, sex, height, body weight, systolic blood pressure (SBP), diastolic blood pressure (DBP), history of hypertension, DM duration. Hypertension was defined as SBP ≥140 mmHg or DBP ≥ 90 mmHg (19). Diagnosis and duration of DM were obtained from medical records. Each participant's DM duration was labeled as those diagnosed for 10 years or more and those who were not. Laboratory parameters included glycated hemoglobin (HbA1c), complete blood count, renal functions such as serum creatinine, urea, UACR, lipid profile including cholesterol, triglyceride, low-density lipoprotein (LDL), and high-density lipoprotein (HDL). Other factors included albumin, total protein (TP), and 25-hydroxyvitamin D. Body mass index (BMI) was calculated using the formula of weight in kilograms divided by height in meters squared (20). The estimated glomerular filtration rate (eGFR) was calculated from serum creatinine using the formula of Chronic Kidney Disease Epidemiology Collaboration (CKD-EPI) equation (21). All blood samples and urine samples were collected in the morning after 8-h fasting before patients taking breakfast.

The eGFR value of all participants was labeled as normal (≥60 mL/min/1.73 m^2^) and impaired (<60 mL/min/1.73 m^2^) according to the definition of CKD (22). The stages of albuminuria was classified with the definition of the USA National Kidney Foundation (23) as normoalbuminuria (UACR <30 mg/g), microalbuminuria (UACR ≥ 30mg/g, <300mg/g), and macroalbuminuria (UACR ≥ 300 mg/g).

### Ophthalmic Examinations

All patients received comprehensive baseline ophthalmic examination, including best-corrected visual acuity (BCVA), intraocular pressure (IOP), dilated fundus examination, fundus photography, and OCT. BCVA was examined with the Snellen chart and converted into the logarithm of minimal angle of resolution (logMAR) (24). IOP was investigated with non-contact tonometry (TX-20 Full Auto Tonometer; Canon, Inc., Tokyo, Japan). All patients received slit lamp fundus examination and fundus photography (TRC-NW8 non-mydriatic) retinal camera, Topcon, Tokyo, Japan; D7500 DSLR camera, Nikon, Tokyo, Japan) to obtained one fovea-centered and one optic nerve head-centered photo after mydriasis. OCT examination (Spectralis; Heidelberg Engineering, Heidelberg, Germany) was conduct to capture the image of the macula, which was composed of 61 B-scans at an automatic real-time (ART) setting (10 images averaged) in the central 30 × 25° area. Central subfield thickness (CST), which was defined as the mean retinal thickness of the 1 mm fovea-centered area, and macular volume (MV), which was the volume of the nine subfields of the Early Treatment Diabetic Retinopathy Study (ETDRS) grid, were obtained automatically from the integrated software. The manual adjustments were performed when there were obvious segmenting or fovea locating errors. Diagnosis and staging of DR and DME were based on the fundus examination, fundus photography, and OCT images follow the International Clinical Diabetic Retinopathy Disease Severity Scale and International Clinical Diabetic Macular Edema Disease Severity Scale by two experienced ophthalmologists specialized in retinal disease (PDR, κ = 0.897; DME, κ = 0.918) (25). Differing diagnoses were reassessed and diagnosed by a senior chief ophthalmologist.

### Body Composition Measurement

All patients underwent BCM examination (InBody S10; InBody CO., LTD., Seoul, Korea), which measured different body compositions via direct segmental multi-frequency bioelectrical impedance analysis (DSM-BIA). An eight-electrode system connecting to the ankles and two fingertips of each patient's hand segments the human body into five parts, including the right arm, left arm, right leg, left leg, and trunk, and measures different body parts compartments. The instrument uses a current of 6 frequencies, including 1 kHz, 5 kHz, 50 kHz, 250 kHz, 500 kHz, and 1 MHz, the varied cell membrane conductivities from high-frequency to low-frequency current are therefore translated into extracellular water (ECW) and intracellular water (ICW) with different impedance (26). In the in-built software, other data including ECW/TBW of the whole body and separate body compartments, including right arm, left arm, trunk, right leg and left leg, and percentage of body fat (PBF), visceral fat area (VFA), skeletal muscle index (SKI) and phase angle (PhA) were calculated. All patients went through the examination in a supine position after 8-h fasting before breakfast, right before the OCT examination.

### Statistical Analysis

Participants were divided into three groups, including DM group (without DR), DR group (diagnosed with DR but no DME), and DME group (diagnosed with DR and DME) according to their diagnosis. Normally distributed continuous variables were shown as mean ± standard deviation in Table 1; χ^2^ tests were carried out to compare non-parametric variables among the three groups. Tests for homogeneity of variance were conducted on the general characteristics, BCM parameters, and renal function parameters, and no variate was found significant. For each participant, the one eye with higher CST, MV, and more severe DR stage was included in the data analysis. Analysis of variance (ANOVA) was applied to compare the quantitative characteristics, including age, SBP, DBP, BMI, BCVA, IOP, blood test parameters, and BCM results among three groups. Two multivariate linear models were carried out to estimate the effect of renal functions on body fluid status among three diabetic groups after adjusting for significant covariates identified in the univariate linear model. Step-by-step multivariate binary logistic regressions with a backward method were conducted to predict DME risk. The models' receiver operating characteristic (ROC) curves were then drawn to demonstrate their predictive power quantified by the area under the curves (AUC). All statistical analysis was performed using SPSS software (version 26.0, IBM Corp., Armonk, NY, USA). A *P* < 0.05 indicates statistical significance.

**Table 1:** General characteristics, systemic parameters and ocular parameters of patients among three groups.

**Characteristics**	**DM, *n* = 35**	**DR, *n* = 31**	**DME, *n* = 66**	***P* ^a^**	***P* ^b^**	***P* ^c^**	***P* ^d^**
Female, *n* (%)	19 (54.3)	17 (54.8)	23 (34.8)	0.075^e^	-	-	-
Hypertension, *n* (%)	11 (31.4)	14 (45.2)	31 (47.0)	0.303^e^	-	-	-
DM duration <10 years, *n* (%)	25 (71.4)	7 (22.6)	27 (40.9)	<0.001^*^^e^	-	-	-
Age, years	56.3 ± 15.5	60.6 ± 8.8	57.7 ± 9.3	0.293	0.380	1.000	0.707
SBP, mmHg	131.8 ± 24.7	133.3 ± 20.7	137.6 ± 18.5	0.358	1.000	0.551	1.000
DBP, mmHg	82.5 ± 14.8	78.6 ± 11.4	81.5 ± 11.6	0.406	0.606	1.000	0.820
MAP, mmHg	98.9 ± 16.7	96.8 ± 13.2	100.2 ± 12.0	0.516	1.000	1.000	0.755
BMI, kg/m^2^	24.9 ± 3.0	24.1 ± 3.4	23.1 ± 2.7	0.013^*^	0.885	0.012^*^	0.324
HbA1c, %	8.5 ± 3.1	8.7 ± 2.0	8.1 ± 1.8	0.620	1.000	1.000	0.572
Hemoglobin, g/L	135.2 ± 15.3	121.8 ± 21.6	120.1 ± 17.5	0.387	0.009^*^	<0.001^*^	1.000
TP, mg/L	69.2 ± 7.4	71.9 ± 5.8	71.6 ± 8.9	0.281	0.525	0.445	1.000
Albumin, g/L	39.8 ± 3.2	40.0 ± 4.7	38.7 ± 5.4	0.381	1.000	0.897	0.673
Urea, mmol/L	5.6 ± 2.1	8.3 ± 4.5	9.1 ± 4.4	<0.001^*^	0.016^*^	<0.001^*^	1.000
Creatinine, μmol/L	68.3 ± 20.0	134.8 ± 166.3	116.4 ± 77.7	0.016^*^	0.020^*^	0.061	1.000
eGFR, mL/min/1.73 m^2^	95.6 ± 24.0	67.3 ± 25.5	68.8 ± 28.4	<0.001^*^	<0.001^*^	<0.001^*^	1.000
UACR, mg/gCr	21.6 ± 29.9	785.3 ± 1640.8	1265.7 ± 1381.4	<0.001^*^	0.057	<0.001^*^	0.271
UACR stage, median (range)^f^	1 (1–2)	2 (1–3)	3 (1–3)	<0.001^*^^e^	-	-	-
25-hydroxyvitamin D, ng/mL	24.7 ± 7.8	17.5 ± 6.9	18.7 ± 7.0	0.023^*^	0.029^*^	0.038^*^	1.000
NLR	2.2 ± 1.2	2.7 ± 1.3	2.9 ± 1.2	0.056	0.422	0.050	1.000
MLR, 10^−1^	2.4 ± 1.2	2.7 ± 1.3	2.9 ± 1.2	0.021^*^	0.515	0.017^*^	0.767
PLR	112.2 ± 43.2	145.4 ± 50.5	149.1 ± 51.1	0.001^*^	0.021^*^	0.001^*^	1.000
**Ocular parameters**							
LogMAR	0.1 ± 0.2	0.4 ± 0.5	0.6 ± 0.4	<0.001^*^	0.085	<0.001^*^	0.010^*^
IOP, mmHg	14.0 ± 3.0	14.0 ± 3.4	13.8 ± 3.8	0.942	1.000	1.000	1.000
**OCT values**							
CST, μm	264.9 ± 20.6	268.0 ± 29.6	417.5 ± 168.1	<0.001^*^	1.000	<0.001^*^	<0.001^*^
MV, mm^3^	8.5 ± 0.4	8.7 ± 0.5	10.9 ± 2.6	<0.001^*^	1.000	<0.001^*^	<0.001^*^
**BCM parameters**							
ECW/TBW, %	38.1 ± 0.7	38.7 ± 0.9	39.2 ± 0.9	<0.001^*^	0.008^*^	<0.001^*^	0.019^*^
PBF, %	29.3 ± 7.3	27.7 ± 5.8	26.3 ± 7.8	0.125	1.000	0.130	1.000
VFA, cm^2^	91.8 ± 35.6	87.9 ± 28.9	78.8 ± 28.5	0.101	1.000	0.131	0.546
BMR, kcal	1386.3 ± 184.2	1336.7 ± 212.2	1354.3 ± 213.9	0.604	0.991	1.000	1.000
SMI, kg/m^2^	7.1 ± 1.1	6.8 ± 1.1	6.9 ± 1.2	0.467	0.766	0.913	1.000
Phase angle	5.7 ± 0.8	5.3 ± 0.8	4.9 ± 0.8	<0.001^*^	0.231	<0.001^*^	0.014^*^

*DM, diabetes mellitus; DR, diabetic retinopathy; DME, diabetic macular edema; SBP, systolic blood pressure; DBP, diastolic blood pressure; MAP, mean arterial pressure; BMI, body mass index; HbA1c, glycated hemoglobin; TP, total protein; eGFR, estimated glomerular filtration rate; UACR, urine albumin-to-creatinine ratio; NLR, neutrophil-to-lymphocyte ratio; MLR, monocyte-to-lymphocyte ratio; PLR, platelet-to-lymphocyte ratio; LogMAR, the logarithm of minimal angle of resolution, for best-corrected visual acuity; IOP, intraocular pressure; CST, central subfield thickness; MV, macular volume; ECW/TBW, extracellular water-to-total body water ratio; PBF, percentage of body fat; VFA, visceral fat area; BMR, basal metabolic rate; SMI, skeletal muscle index*.

a *, b, c, dwere acquired using one-way ANOVA*;

e * was acquired using χ^2^ tests*.

*^a, e^P-value among three group*.

b *P-value between DM group and DR group*.

c *P-value between DM group and DME group*.

d *P-value between DR group vs DME group*.

*^d^UACR stage including normoalbuminuria, microalbuminuria and macroalbuminuria were denoted as stage 1 to 3*.

* *Stand for P <0.05*.

## Results

One hundred thirty-two patients (73 male and 59 female) with 132 eyes were enrolled in the study. Table 1 shows the details of general characteristics, systemic factors, ocular parameters and BCM parameters among the DM group (*n* = 35), DR group (*n* = 31), and DME group (*n* = 66). As for general characteristics, there was no statistically significant difference in sex, age, hypertension, blood pressure, weight among the three groups (all *P* > 0.05), and there were significant differences in DM duration (*P* < 0.001) and BMI (*P* = 0.013). BCVA, CST and MV were significantly different among three groups (all *P* < 0.001) while IOP was not (*P* = 0.942).

### Systemic Factors and BCM Parameters Among Three Groups

All parameters of blood test and BCM parameters were found normally distributed. In the test of homogeneity, PhA and ECW/TBW showed a high level of collinearity. Hence PhA was excluded in further analysis. In the *post-hoc* analysis, systemic parameters including urea and PLR in both the DME group and DR group were significantly higher than the DM groups (all *P* < 0.05), while hemoglobin, 25-hydroxyvitamin D, eGFR were significantly lower than the DM group (all *P* < 0.05). UACR (DME group: 1265.7 ± 1381.4 mg/gCr; DM group: 21.6 ± 29.9 mg/gCr, *P* < 0.001) and MLR (DME group: 2.9 ± 1.2 × 10^−1^; DM group: 2.4 ± 1.2 × 10^−1^, *P* = 0.017) was found elevated in the DME group compared to DM group. As for BCM parameters, the DME group had significantly higher ECW/TBW than both the DM group and DR group (*P* < 0.001). No significant differences of PBF, VFA, BMR, and SMI were detected among three groups (all *P* > 0.05).

### Association Between DME and Fluid Overload

Table 2 demonstrates the association between DME and fluid overload. Univariate linear model (Model 1) shows that DME (compared to both DR and DM), age, DM duration, and UACR were positively associated with fluid overload, while albumin level and eGFR were negatively associated with fluid overload (all *P* < 0.05). No significant association was found between MAP and ECW/TBW (*P* = 0.188). In the multivariate linear model, DME, DR, age, MAP, and albumin showed significant impact on ECW/TBW (all *P* < 0.05; Model 2), while eGFR was not significantly associated with ECW/TBW (*P* = 0.620; Model 2). In the multivariate model focusing on UACR, only DME, age, and UACR were positively associated with ECW/TBW (all *P* < 0.05), while MAP showed a negative association with ECW/TBW (*P* = 0.005).

**Table 2:** Influence factors of ECW/TBW with univariate and multivariate linear models.

	**Model 1**				**Model 2**				**Model 3**			
	**β**	**95%CI**	**Exp β**	***P***	**β**	**95%CI**	**Exp β**	***P***	**β**	**95%CI**	**Exp β**	***P***
**Groups**
DM	Reference	–	–	–	Reference	–	–	–	Reference	–	–	–
DR	0.627	0.229–1.026	1.873	0.002^*^	0.526	0.123–0.929	1.692	0.011^*^	0.387	0.036–0.809	1.472	0.073
DME	1.131	0.793–1.469	3.099	<0.001^*^	1.095	0.765–1.425	2.989	<0.001^*^	0.883	0.530–1.236	2.418	<0.001^*^
**Groups**
DR	Reference	–	–	–	Reference	–	–	–	Reference	–	–	–
DME	0.504	0.152–0.855	1.656	<0.001^*^	0.569	0.268–0.869	1.767	<0.001^*^	0.496	0.196–0.796	1.642	0.001^*^
Age	0.037	0.024–0.05	1.038	<0.001^*^	0.038	0.026–0.049	1.038	<0.001^*^	0.038	0.027–0.048	1.039	<0.001^*^
DM Duration	0.160	0.005–0.315	1.173	0.043^*^	−0.057	−0.185–0.072	0.945	0.389	−0.006	−0.137–0.125	0.994	0.926
MAP	−0.008	−0.02–0.004	0.992	0.188	−0.011	−0.019 to −0.002	0.990	0.017^*^	−0.012	−0.021 to −0.004	0.988	0.005^*^
Albumin	−0.051	−0.084–0.018	0.950	0.003^*^	−0.046	−0.073 to −0.02	0.955	0.001^*^	−0.018	−0.052–0.016	0.982	0.296
eGFR	−0.013	−0.018–0.008	0.987	<0.001^*^	−0.001	−0.006–0.004	0.999	0.620	–	–	–	–
UACR	0.030	0.019–0.041	1.031	<0.001^*^	–	–	–	–	0.017	0.004–0.029	1.017	0.010^*^

*DM, diabetes mellitus; DR, diabetic retinopathy; DME, diabetic macular edema; MAP, mean arterial pressure; eGFR, estimated glomerular filtration rate; UACR, urine albumin-to-creatinine ratio*.

*DM duration, every 5 years; MAP, per mmHg; Albumin, per g/L; eGFR, per mL/min/1.73 m^2^; UACR, per 10^−2^ mg/g*.

*Model 1: univariate analysis; Model 2: multivariate model adjusted for systemic covariates and eGFR; Model 3: multivariate model adjusted for systemic covariates and UACR*.

*P was acquired using generalized linear model*.

* *Stands for P <0.05*.

### Fluid Overload and Its Related Influence Factors of DME

**Figure 1:**
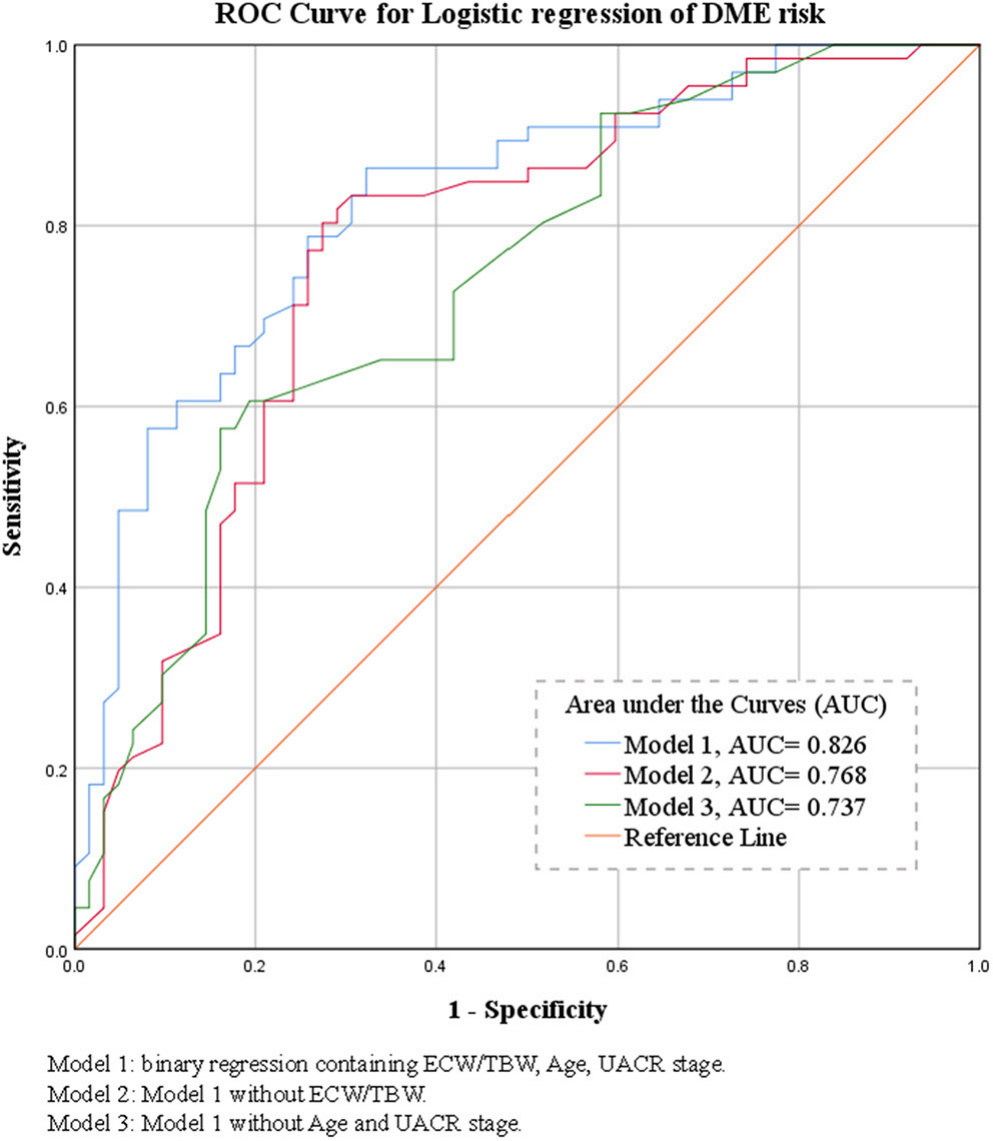
Binary logistic regression receiver operating characteristic curves of the morbidity of DME and their areas under the curve.

In the binary logistic regression analysis with backward method of the risk of DME, age (per 1 year, OR = 0.941, *P* = 0.009), UACR stage (microalbuminuria, OR = 5.16, *P* = 0.005; macroalbuminuria, OR = 5.198, *P* = 0.003), and ECW/TBW (per 10^−2^, OR = 2.814, *P* = 0.002) entered the model under the probability for removal at 0.05. Details were shown in Table 3. The predictive power of the multivariate regression models was demonstrated as ROC curves shown in Figure 1 (Model 1: ECW/TBW, UACR, and Age: AUC = 0.826; Model 2: UACR and Age: AUC = 0.768; Model 3: ECW/TBW: AUC = 0.737).

**Table 3:** Multivariate binary logistic regression for DME risk.

	**β**	**S.E**.	***P***	**Exp (β)**
ECW/TBW	1.035	0.327	0.002^*^	2.814
Age	−0.06	0.023	0.009^*^	0.941
Normoalbuminuria	Reference	-	-	-
Microalbuminuria	1.641	0.588	0.005^*^	5.16
Macroalbuminuria	1.648	0.55	0.003^*^	5.198

*DME, diabetic macular edema; ECW/TBW, extracellular water-to-total body water ratio*.

*Adjusted r^2^ of logistic regression model = 0.401*.

*Exp (β) stands for standardized regression coefficient*.

* *Stands for P <0.05*.

## Discussion

This study provided new evidence to demonstrate the role of fluid overload in DME. As previously described, the relationship between barrier dysfunction and macular edema, impaired renal function and fluid overload have been investigated, respectively (10, 15). However, the complicated interaction between barrier dysfunction and fluid overload has not been elucidated yet, which make it intricate to explain whether the fluid overload is the mediator between barrier dysfunction and macular edema, or it can lead to DME without the impact from BRB breakdown, as fluid overload also had other influencing factors (27–29). Our study corroborated that fluid overload existed in patients with DME. Furthermore, we were the first to discover that the relationship between fluid retention and DME is independent of renal functions, especially UACR and eGFR. ECW/TBW was proven to be a risk factor for DME, suggesting that fluid overload is a contributing factor to DME apart from BRB disruption.

Tsai et al. (15) had found that the level of fluid overload was correlated with CST in patients with DR, and volume overload was the influence factor of DME. Our study also found a significant difference in fluid status between DME patients and DM patients without DME, either with or without DR. These results suggested that volume expansion might contribute to DME development. Meanwhile, we did not find significant differences in fluid overload status between the DR and DM groups, indicating that fluid overload could be a risk factor for DME but not DR.

A recent study found that fluid overload was correlated with the stages of UACR in T2DM patients with DKD (14). DKD was regarded as a sign of endothelial dysfunction due to oxidative stress and inflammation (30). Also, multiple studies confirmed that UACR levels were significantly associated with DME (6, 7, 31). Hsieh et al. (32) found patients with higher baseline UACR levels to have higher risks for developing DME during the follow-up period than those with lower UACR levels. Nephropathy and retinopathy co-existed in diabetic patients (33). Hemodynamic and structural changes due to inflammatory cytokines and oxidative stress under high blood glucose have been speculated as the cause (31). The increased UACR level suggests albumin leakage in the glomerulus and indicates the vascular barrier breakdown systemically. Even though the BRB was formed predominantly with tight junctions different to peripheral capillaries (34), in our study, the UACR stages were associated with ECW/TBW after adjustment, indicating that UACR is a significant influence factor for fluid overload. Therefore, it was of great importance to consider the role of DKD presented by albuminuria in the process of fluid retention in DME.

As ECW/TBW was reported to be affectable by multiple factors (14, 35, 36), we gradually adjusted for systemic factors, eGFR, and albuminuria, when analyzing fluid status among all participants. DME and ECW/TBW were still positively related after adjusting for confounders, suggesting that in DM patients, DME happens simultaneously with the presence of fluid overload, which was not affected by apparent inner BRB breakdown. Our results confirmed that the two parameters, ECW/TBW and UACR, are independent risk factors for DME. Hence, it could be speculated that the role of systemic fluid overload in DME development may collaborate with other mechanisms than deteriorated BRB function.

Throughout the body, fluid volume, hydrostatic and oncotic pressure, levels of albumin, and capillary permeability are the keys to maintaining the fluid balance between the intracellular and extracellular spaces, which is also true in the retina case. One of the popular theories for DME development was explicated by the starling equation (13). The hydrostatic pressure and oncotic pressure in different compartments govern the movement direction of fluid, which maintain homeostasis under normal circumstances (37). For example, the water transport of RPE cells was proved coupling with lactate, which provide a positive oncotic pressure to drive the water flow (38). It can be speculated that when hydrostatic pressure increases in the capillaries under volume expansion, fluid in the capillaries tends to move to the extravascular space (13). Therefore, high fluid volume can lead to peripheral edema and macular edema in the eyes. Recently, the revised starling equation has risen to make up for the shortcoming of the traditional one (37). The vascular barrier was composed of not only endothelial cells but also a layer which is known as the endothelial surface layer (ESL). ESL is the inner surface of the vascular wall, a semi-permeable layer composed of the glycocalyx and plasma protein such as albumin. Especially, albumin plays a vital role in its filtration function. ESL is usually impaired under systemic inflammation, including DM. The increased production of acute-phase protein suppresses albumin synthesis in the liver due to the limited synthesis capacity. Moreover, increased excretion of albumin from the kidney in the long run in patients with impaired kidney function further decreases the albumin level, leading to the dysfunction of ESL and leakage of fluid and protein. While on the retina, the damaged Müller cells no longer sustain neovascular coupling with DR progression, creating a focal high permeable environment for the albumin (39). Taking together, both fluid overload and impaired blood-retinal barrier can lead to leakage of fluid into the retinal interstitial space, resulting in macular edema.

As retinal thickness decreases with aging (40), we found the model consisting of ECW/TBW, UACR, and age achieved high accuracy in predicting DME risk. This result tally with the hypothesis that fluid overload could be a systemic fluid marker, parallel to the barrier dysfunction represented by albuminuria in the development of DME, in addition to focal VEGF elevation and inflammation.

In clinical practice, anti-VEGF agents are considered the first-line treatment for DME. However, non-responders exits at a considerable percentage (41). Some cases are even refractory to both anti-VEGF treatment and corticosteroid (42), demonstrating the need for additional therapies to treat this disease more comprehensively. Also, it is known that DME does not necessarily fit the regular course of DR progression. It may occur at any stage of DR (43). Therefore, beyond ischemia and inflammation, increased fluid volume may be another critical component in the pathophysiology of DME that has not been given sufficient attention. Several researchers also reported cases in which patients with DME improved, both central retinal thickness reduced and visual acuity improved, after diuretic therapy and restricting salt intake (44, 45), some of whom were even resistant to ophthalmic intervention anti-VEGF and grid laser treatment (46). Considering our findings in this study, we propose that, on the one hand, fluid overload can be one of the mechanisms that cause DME. In this point, treatment to ameliorate extracellular volume expansion such as diuretics and sodium-glucose cotransporter 2 inhibitors may have a role in treating DME patients with high ECW/TBW. On the other hand, in cases of DME who inadequately respond to anti-VEGF therapies and anti-inflammatory drugs, the underlying disease pathology may be mediated by fluid retention. Hence, further study with longitudinal design to observe the treatment response of DME patients with different fluid statuses would be pivotal to confirm the hypothesis.

There were some limitations of the study. The sample size was calculated with preliminary study data. Still, more substantial statistical power might reveal more significant systemic influence factors. Also, the assessment of the risk of DME was to be tested prospectively, and further cohort studies should be conducted to collaborate with our results.

In conclusion, systemic fluid status elevated significantly in patients with DME compared with diabetic patients without DME. Fluid overload and albuminuria were independent risk factors for DME. Fluid overload might have initiated the extravasation in macula edema in addition to barrier dysfunction.

## Data Availability Statement

The raw data supporting the conclusions of this article will be made available by the authors, without undue reservation.

## Ethics Statement

The studies involving human participants were reviewed and approved by Research Ethics Committee of the Guangdong General Hospital (GDREC2018380H). The patients/participants provided their written informed consent to participate in this study.

## Author Contributions

JY, QP, and YL contributed to the study conception and design. Material preparation and data collection were performed by AL, JX, XZ, RC, YC, and ZW. Data analysis was conducted and the first draft of the manuscript was written by JY and QP. DC and LZ contributed to the manuscript reviewing and editing and funding acquisition. All authors read and approved the final manuscript.

## Funding

This work was funded by Guangzhou Municipal Science and Technology Bureau (grant 202102080008) and Bethune-Merck Diabetes Research Fund (G2018030).

## Conflict of Interest

The authors declare that the research was conducted in the absence of any commercial or financial relationships that could be construed as a potential conflict of interest.

## Publisher's Note

All claims expressed in this article are solely those of the authors and do not necessarily represent those of their affiliated organizations, or those of the publisher, the editors and the reviewers. Any product that may be evaluated in this article, or claim that may be made by its manufacturer, is not guaranteed or endorsed by the publisher.

## Abbreviations

DM: diabetes mellitus
DR: diabetic retinopathy
DME: diabetic macular edema
SBP: systolic blood pressure
DBP: diastolic blood pressure
BMI: body mass index
LogMAR: logarithm of minimal angle of resolution
BCVA: best-corrected visual acuity
IOP: intraocular pressure
CST: central subfield thickness
MV: macular volume
UACR: urine albumin-to-creatinine ratio
eGFR: estimated glomerular filtration rate
HbA1c: glycated hemoglobin
TP: total protein
HDL: high-density lipoprotein
LDL: low-density lipoprotein
NLR: neutrophil-to-lymphocyte ratio
MLR: monocyte-to-lymphocyte ratio
PLR: platelet-to-lymphocyte ratio
PBF: percentage of body fat
ECW/TBW: extracellular water-to-total body water ratio
VFA: visceral fat area
BMR: basic metabolic rate
SMI: skeletal muscle index
DKD: diabetic kidney disease
VEGF: vascular endothelial growth factor
BIA: bioimpedance analysis
BCM: body composition measurement
CKD: chronic kidney disease
OCT: optical coherence tomography
ART: automatic real time
DSM-BIA: direct segmental multi-frequency bioelectrical impedance analysis
ECW: Extracellular water
ICW: intracellular water
PhA: phase angle
CKD-EPI: Chronic Kidney Disease Epidemiology Collaboration
ESL: endothelial surface layer
IVI, intravitreous injection, RPE: retinal pigment epithelium
BRB: blood-retinal barrier.
